# Hypoxic and nitrosative stress conditions modulate expression of myoglobin genes in a carcinogenic hepatobiliary trematode, *Clonorchis sinensis*

**DOI:** 10.1371/journal.pntd.0009811

**Published:** 2021-09-30

**Authors:** Seon-Hee Kim, Dongki Yang, Young-An Bae

**Affiliations:** 1 Department of Microbiology, Lee Gil Ya Cancer and Diabetes Institute, Gachon University College of Medicine, Incheon, Republic of Korea; 2 Department of Physiology, Lee Gil Ya Cancer and Diabetes Institute, Gachon University College of Medicine, Incheon, Republic of Korea; National University of Ireland Galway, IRELAND

## Abstract

Despite recent evidence suggesting that adult trematodes require oxygen for the generation of bioenergy and eggshells, information on the molecular mechanism by which the parasites acquire oxygen remains largely elusive. In this study, the structural and expressional features of globin genes identified in *Clonorchis sinensis*, a carcinogenic trematode parasite that invades the hypoxic biliary tracts of mammalian hosts, were investigated to gain insight into the molecules that enable oxygen metabolism. The number of globin paralogs substantially differed among parasitic platyhelminths, ranging from one to five genes, and *the C*. *sinensis* genome encoded at least five globin genes. The expression of these *Clonorchis* genes, named *CsMb* (*CsMb1—CsMb3*), *CsNgb*, and *CsGbX*, according to their preferential similarity patterns toward respective globin subfamilies, exponentially increased in the worms coinciding with their sexual maturation, after being downregulated in early juveniles compared to those in metacercariae. The CsMb1 protein was detected throughout the parenchymal region of adult worms as well as in excretory-secretory products, whereas the other proteins were localized exclusively in the sexual organs and intrauterine eggs. Stimuli generated by exogenous oxygen, nitric oxide (NO), and nitrite as well as co-incubation with human cholangiocytes variously affected globin gene expression in live *C*. *sinensis* adults. Together with the specific histological distributions, these hypoxia-induced patterns may suggest that oxygen molecules transported by CsMb1 from host environments are provided to cells in the parenchyma and intrauterine eggs/sex organs of the worms for energy metabolism and/or, more importantly, eggshell formation by CsMb1 and CsMb3, respectively. Other globin homologs are likely to perform non-respiratory functions. Based on the responsive expression profile against nitrosative stress, an oxygenated form of secreted CsMb1 is suggested to play a pivotal role in parasite survival by scavenging NO generated by host immune cells via its NO dioxygenase activity.

## Author summary

Trematode parasites that invade mammalian tissues have long been believed to produce bioenergy via anaerobic respiration in their definitive hosts. However, recent studies have revealed that these parasites require considerable amounts of oxygen for the generation of hard eggshells during sexual reproduction as well as energy metabolism. Despite these findings, information on the biological mechanisms and relevant molecules responsible for oxygen uptake in the host environment remains largely elusive. *Clonorchis sinensis* is a carcinogenic trematode parasite that causes clonorchiasis in humans by infecting the bile ducts. Here, we investigated globin genes/proteins in the liver fluke. The genome of *C*. *sinensis* encoded at least five globin paralogs (*CsMb1*, *CsMb2*, *CsMb3*, *CsNgb*, and *CsGbX*). Temporal expression of these globin genes coincided with the sexual maturation of *C*. *sinensis*. Based on the histological localities and induction profiles upon hypoxia, it could be postulated that the oxygen molecules transported by CsMb1 from host environments are provided to cells in the parenchyma and intrauterine eggs/sex organs of the worms by CsMb1 and CsMb3, respectively, for energy metabolism and eggshell formation. Other globin homologs were likely to perform non-respiratory functions. In addition, the oxygenated form of secreted CsMb1 seemed to participate in the scavenging of nitric oxide generated by host immune cells via its nitric oxide dioxygenase activity to increase the survival of the parasite.

## Introduction

*Clonorchis sinensis* is a digenean trematode that invades the bile ducts of mammalian hosts and causes clonorchiasis in humans. Humans contract the parasitic infection by eating raw or undercooked freshwater fish containing the metacercariae of the liver fluke. Clonorchiasis is highly prevalent in East Asian countries such as China, Vietnam, and Korea [1]. The spectra of clinical manifestations and signs of acute clonorchiasis are variable and range from asymptomatic infection to mild febrile illness accompanied by right upper quadrant pain and intermittent colic pain, depending on the worm burden. Alternatively, chronic clonorchiasis resulting from persistent worm infection induces fibrosis of the ducts, destruction of the adjacent liver parenchyma, and recurrent pyogenic cholangitis. In the worst-case scenario, these histopathological changes eventually lead to the development of cholangiocarcinoma with a significantly poor prognosis [2–4].

The newly excysted metacercariae of *C*. *sinensis* settle down in the hepatobiliary tracts of mammalian hosts [5]. Owing to the extremely low oxygen concentration of the bile duct lumen [6], *C*. *sinensis* and other trematodes such as *Opisthorchis viverrini* and *Fasciola* spp. that invade the biliary ducts are intuitively assumed to perform anaerobic respiration during their juvenile and adult life stages [7]. However, recent investigations have demonstrated that a handful of genes involved in aerobic energy metabolism, such as the citrate cycle and oxidative phosphorylation, are abundantly expressed in *C*. *sinensis* and *O*. *viverrini* adults. The levels of proteins involved in catabolic pathways with a high biochemical oxygen demand, such as β-oxidation of fatty acids, were also high in these liver flukes [8–10]. Furthermore, eggshell proteins of trematode parasites are linked to one another via an oxygen-dependent, quinone-tanning/sclerotization process [11,12]. Taken together, these observations suggest that oxygen is indispensable for the survival and reproduction of these trematodes, and thus, the worms might have evolved specific molecular machinery to satisfy the high oxygen demand in the hypoxic host environment. Globin proteins, including myoglobin with much greater affinity for oxygen than mammalian homologs [13,14], are plausible candidates for the machinery in these trematode parasites.

Globins are small oxygen-binding hemoproteins that play major roles in oxygen transport and facilitate the diffusion of oxygen to the mitochondria [15,16]. Globins and other globin domain-containing proteins are identifiable in almost all living organisms [15]. In gnathostomes, globin homologs are categorized into multiple subfamilies, including androglobin (Adgb), neuroglobin (Ngb), globin X (GbX), hemoglobin (Hb), myoglobin (Mb), and cytoglobin (Cygb), some of which show mosaic distributions across the animal taxa [17,18]. Phylogenetic investigations have demonstrated that the major globin subfamilies are products of vertebrate-specific gene duplication events, followed by structural diversification and lineage-specific losses or expansions. However, the *Adgb*, *Ngb*, and *GbX* genes seemed to have originated prior to the divergence between deuterostomes and protostomes [15,17–19]. In addition to their roles as regulators of mitochondrial oxygen supply, these globins, especially those belonging to the Adgb, Ngb, and Cygb subfamilies, perform functions related to redox-regulated signaling, oxygen sensing, and reactive oxygen species (ROS)/reactive nitrogen species (RNS) detoxification [17,18].

The evolutionary history of the globin gene family was examined in the phylum Nematoda [20,21]. Ancestral globin genes appeared to have undergone a series of gene duplication events specific to the animal clade. The resulting paralogs with unexpectedly high copy numbers were subjected to processes of structural and functional diversification, which were accompanied by cell/tissue-specific expression patterns of respective proteins [20,22]. The clade-specific radiation of globin genes has also been recognized in fungi [23]. The phylum Platyhelminthes represents a large and diverse group of organisms, a significant fraction of which alternates between free-living and parasitic life modes. Based on the complex life modes of platyhelminths in the context of oxygen availability, globin genes are likely to have experienced unique evolutionary events, which may be closely associated with the establishment of the parasitic life mode. Nevertheless, little is known regarding the phylogeny of globin genes and their specific responsive expression to exogenous environmental factors in platyhelminths including parasitic trematodes.

In this study, we identified globin genes in the GenBank databases of *C*. *sinensis* and other representative platyhelminths to examine their unique evolutionary episodes. Together with structural features and spatiotemporal expression patterns, the induction profiles of *C*. *sinensis* genes were determined in worms challenged with different oxygen concentrations. The regulatory effects of nitric oxide (NO) and nitrite, which can modulate the levels of oxy- and deoxy-form Mbs, were investigated in *C*. *sinensis* adults incubated under appropriate experimental conditions. Finally, the probable competition between the parasite and host cells to use oxygen was examined by monitoring the changes in globin gene expression in worms co-incubated with human cholangiocytes.

## Materials and methods

### Ethics statement

The protocols for *C*. *sinensis* infection, maintenance of the animals, and recovery of the parasite under anesthesia were approved by the Institutional Review Board of Gachon University, Korea (protocol numbers GIACUC-R2015004 and LCDI-2020-0136). The animals were housed in accordance with the guidelines of the Association for the Assessment and Accreditation of Laboratory Animal Care (Thailand). The use of animals during the preparation of specific mouse antisera was approved by the Institutional Review Board (protocol number GIACUC-R2015003).

### *In silico* identification of *C*. *sinensis* Mb proteins

Globin sequences that represent each of the multiple globin subfamilies were isolated from GenBank (https://www.ncbi.nlm.nih.gov/) based on previous reports [17,19,24]. These sequences were used as queries to identify homologous proteins in the *C*. *sinensis* proteomic database using BLASTp. Trematode and nematode proteins as well as those of humans and zebrafish, annotated as one of the globin subfamilies, were also retrieved from the databank. The protein sequences were examined using the SMART program (http://smart.embl.de/) to define an amino acid block corresponding to the tightly conserved globin domain. After removing redundant entries, the globin-domain sequences of 151 proteins were used in the construction of a guide tree using Clustal X (S1 Fig).

### Structural and phylogenetic analyses

Using the amino acid sequences of *C*. *sinensis* and human globins, the genomes, transcriptomes, and proteomes of selected trematodes (*O*. *viverrini* and *Fasciola hepatica*, https://www.ncbi.nlm.nih.gov/; *Schistosoma mansoni*, https://www.ncbi.nlm.nih.gov/ and https://parasite.wormbase.org/) and cestodes (*Echinococcus granulosus*, *Echinococcus multilocularis*, *Hymenolepis microstoma*, and *Taenia solium*; http://www.genedb.org/), of which whole genomic information is available, were comprehensively examined to identify globin orthologs. *Schmidtea mediterranea* databases (Turbellaria; http://smedgd.neuro.utah.edu/ and https://www.ncbi.nlm.nih.gov/) were also used for the screening. The amino acid sequences of the retrieved proteins were aligned with those of the human and zebrafish globins using MUSCLE (https://www.ebi.ac.uk/Tools/msa/muscle/) and manually trimmed using GeneDoc [25]. The sequence alignment (35 sequences; 146 amino acid positions) was used to construct a maximum-likelihood tree using PhyML (Le and Gascuel model for amino acid evolution, equilibrium frequencies of 20 amino acids observed in the data set, gamma distributed rates among sites, and pairwise deletion of missing data) [26]. Branch support was inferred using the non-parametric Shimodaira-Hasegawa-like approximate likelihood ratio test (SH-aLRT) provided by PhyML. The resulting tree was displayed by TreeView [27].

The exon-intron architectures of the globin genes were determined by comparing the nucleotide sequences between complementary/coding DNAs (cDNAs/CDSs) and corresponding genomic DNA segments, which were isolated from each of the respective databanks. The insertion phases of introns relative to the reading frame were defined as follows: 0, insertion between codons; +1, insertion between the first and second bases of a codon; and +2, insertion between the second and third bases of a codon. Introns with identical insertion phases and intervening sites, which were verified by checking their positions in the amino acid sequence alignment, were considered orthologous.

### Parasitic materials

*C*. *sinensis* metacercariae obtained from freshwater fish in a clonorchiasis-endemic area of Korea were directly administered into the stomachs of Sprague-Dawley rats by oral gavage (150 metacercariae/rat). The parasitic worms were collected from the bile ducts of the experimental animals at regular intervals from 2 to 56 days post-infection.

The live *C*. *sinensis* metacercariae or worms were washed several times with ice-cold physiological saline and incubated in RPMI-1640 medium (phenol red- and serum-free, pH 7.2) containing antibiotics at 37°C in a 5% CO_2_ incubator. The medium was replaced with fresh RPMI-1640 after 1 h and incubated for an additional 2 h. The incubation medium was centrifuged at 3,000 rpm for 10 min and subsequently at 10,000 × *g* for 30 min at 4°C. The final supernatant was taken as the excretory-secretory product (ESP) of *C*. *sinensis*. *C*. *sinensis* worms were homogenized in RIPA buffer (pH 7.4; 10 mM Tris-HCl, 150 mM NaCl, 1% Nonidet P-40, 0.2% sodium deoxycholate, 1 mM EDTA, and 10 mM NaF) using a Dounce tissue grinder (Wheaton, Millville, NJ, USA). The homogenates were centrifuged at 10,000 × *g* for 30 min at 4°C. Total proteins in the supernatants and ESP were quantified using the Pierce BCA Protein Assay Kit (Thermo Fisher Scientific, Rockford, IL, USA). The protein and ESP samples were stored at -80°C until use.

### Preparation of recombinant proteins and specific antisera

Full CDSs of the *C*. *sinensis* globin genes were amplified from adult worm cDNAs using gene-specific primers containing restriction sites for *Eco*R I and *Xho* I (*Bam*H I and *Hin*d III in primers for *CsNgb*; S1 Table). After digestion with the corresponding endonucleases, the PCR products were ligated into the pET-28a vector (Novagen, Madison, WI, USA). The plasmid constructs were transformed into competent *Escherichia coli* DH5α cells, and their expression fidelity was confirmed by sequencing. The plasmids were introduced into *E*. *coli* BL21 (DE3) cells. Expression of the *C*. *sinensis* genes was induced in Luria-Bertani medium containing 0.5 mM isopropyl-β-D-thiogalactopyranoside (IPTG) for 4 h at 37°C. The bacterial cells were sonicated and the recombinant globin proteins were purified under native conditions using nickel-nitrilotriacetic acid (Ni-NTA) and agarose chromatography (Qiagen, Valencia, CA, USA). The purity of the eluents was examined by 12% SDS-PAGE under reducing conditions and quantified using the Pierce BCA protein assay kit.

Each recombinant protein (30 μg) was mixed with Freund’s adjuvants (Sigma-Aldrich, St. Louis, MO, USA) and subcutaneously injected into specific pathogen-free BALB/*c* mice three times at 2-week intervals. The mice were finally boosted with 10 μg of protein through the tail vein and were sacrificed 7 days later. Blood samples collected through heart puncture were centrifuged for 10 min at 3,000 × *g* and 4°C. The supernatants were used as mouse antisera specific to *C*. *sinensis* globins. The specific reactivity of these antisera was cross-checked with the recombinant proteins (S2 Fig) and used for western blotting and immunohistochemical staining of native homologs.

### Ontogenic profiling of globin gene expression

Total RNA was extracted from *C*. *sinensis* worms at various developmental and maturation stages (> 30 worms/stage) using the QIAzol solution and an RNeasy mini kit (Qiagen, Hilden, Germany). After removing any contaminating DNA with RNase-free DNase (New England Biolabs, Ipswich, MA), the RNA samples were used for cDNA synthesis with the iScript cDNA synthesis kit (Bio-Rad, Munich, Germany). The relative levels of globin gene transcripts in the cDNA solutions were examined using quantitative real-time PCR (qPCR) with gene-specific primers (S1 Table). To ensure amplification of the corresponding cDNA segments, the primer pairs were designed from the nucleotide sequences of different exons flanking a long-intervening intron. A gene encoding *C*. *sinensis* β-actin (GAA53474) was selected as a reference in this quantitative analysis [28]. The qPCR was performed using SYBR Green Master Mix and the CFX96 detection system following the manufacturer’s instructions (Bio-Rad Laboratories, Hercules, CA). Melt curve analysis was performed to assess the presence of a single amplicon. The reactions were conducted in triplicate and the results were presented as the mean ± standard deviation (SD). The relative expression levels of globin genes were calculated against the reference β-actin gene ($2^{-\Delta {\text{C}}_{\text{T}}}$ method) [29]. Pairwise comparisons between the means of different groups were performed using the Student’s *t-*test (two-tailed test; *P*-value < 0.05).

### Effects of oxygen, nitrite, nitrate, and bile on the expression of globin genes

Live 28-day-old *C*. *sinensis* worms (10 worms/group) were incubated in RPMI-1640 medium (phenol red- and serum-free, pH 7.2) at 37°C in 5% CO_2_ incubators. Using nitrogen gas, the oxygen level in the incubator was equilibrated to 1%, 5%, or 20%. In a series of experimental groups, the incubation medium was supplemented with sodium nitrite (5 and 20 μM; Sigma-Aldrich), sodium nitrate (5 and 20 μM; Sigma-Aldrich), or bile salts (0.04%; Sigma-Aldrich, Oakville, ON, Canada). After 24 h incubation, worms of each experimental group, which had been prepared in triplicate, were mixed together and used for the extraction of mRNAs to reduce errors associated with inter-individual variability. Fold changes in the expression of globin genes were calculated for the unincubated *C*. *sinensis* worms ($2^{-\Delta \Delta {\text{C}}_{\text{T}}}$ method) [29] via qPCR analysis of the mRNAs as described above.

### Chemical irritation of *C*. *sinensis* worms with nitric oxide (NO)

The kinetics of NO released by an NO donor, NOR-4 (Enzo Life Sciences, Farmingdale, NY, USA) was determined by incubating RPMI-1640 medium containing the chemical (50 and 200 μM) and 4,5-diaminofluorescein (DAF-2, 10 μM; Enzo Life Sciences) for 24 h at 37°C in a 5% CO_2_ incubator with atmospheric oxygen tension. The fluorescence of the incubating media was periodically measured at room temperature using a spectrofluorometer (excitation and emission wavelengths, 495 nm and 515 nm, respectively) [30].

The 28-day-old *C*. *sinensis* adults (15 worms/well) were incubated in RPMI 1640 medium supplemented with NOR-4 and DAF-2 at 37°C under 1% and 20% oxygen conditions. After 24 h incubation, the conditioned media and worm bodies were used for the measurement of fluorescence and the extraction of total RNA, respectively. The RNA samples were used to analyze globin gene expression using qPCR. Concurrently, experiments with dead worms, which were prepared by heating the worms at 55°C for 30 min, were used as comparative control groups. All the experiments were performed in triplicate and worms from each experimental group were collectively used for mRNA extraction.

### Co-incubation of human cholangiocyte and *C*. *sinensis*

The human cholangiocyte H69 cells seeded on six-well plates (2 × 10^5^ cells/well) were incubated in DMEM/HamF-12 medium (Gibco BRL) supplemented with 10% FBS, 100 U/mL penicillin, 100 μg/mL streptomycin, 0.18 mM adenine, 5 μg/mL insulin, 5.5 μM epinephrine, 2 nM triiodothyronine, 5 μg/mL transferrin, 1.64 μM epidermal growth factor, and 1.0 μM hydrocortisone. When the cultures reached approximately 80% confluence, polycarbonate transwell inserts (pore size, 3 μm; SPL Life Sciences, Pochen, Korea) containing live 28-day-old *C*. *sinensis* adults (15 worms/well) were placed into the culture plates. The plates were further incubated at 37°C for 24 h in 5% CO_2_ incubators under 1% and 20% oxygen conditions. During co-incubation, nitrite was added to a series of wells. Total RNAs extracted from the *C*. *sinensis* worms were used for the analysis of globin gene expression using qPCR. RNAs from H69 cells were also used to examine human hypoxia-inducible factor 1α (*HIF-1α*) and *HIF-2α* expression using the primers as previously described [31] (S1 Table). All the experiments were performed in triplicate, and worms or cells of each experimental group were collectively used for the mRNA extraction. The experiment was repeated once more by using 56-day-old worms under the same conditions.

### Western blot analysis

The *C*. *sinensis* proteins in the whole body extracts (30 μg) and ESP (10 μg) were resolved on 12% SDS-PAGE gels under reducing conditions and transferred onto nitrocellulose membranes (Schleicher & Schuell Bioscience, Dassel, Germany). The protein blots were reacted with mouse antisera specific to each of the *C*. *sinensis* globins (1:1,000 dilution) and then with horseradish peroxidase-conjugated rabbit anti-mouse IgG antibody (Bethyl Laboratories, Montgomery, TX, USA). Positive signals were developed with enhanced chemiluminescence detection reagents (GE Healthcare Life Science, Pittsburgh, PA, USA) and visualized on X-ray films (Fujifilm, Tokyo, Japan).

### Immunohistochemistry

Paraffin blocks containing the whole bodies of *C*. *sinensis* metacercariae or adults were cut into 4 μm slices on electrostatically charged glass slides (Superfrost Plus, Manzel-Glaser, Germany). After deparaffinization and rehydration according to the manufacturer’s instructions (DAKO, Carpinteria, CA, USA), the sections were treated with 3% H_2_O_2_ for 5 min and blocked with 3% BSA in TBS/T buffer for 1 h. The slides were incubated overnight at 4°C with mouse antisera specific to the *C*. *sinensis* recombinant globins (1:200 dilution in TBS/T supplemented with 3% BSA) and then with horseradish peroxidase (HRP)-conjugated goat anti-mouse IgG antibody (1:1,000 dilution, Cappel). HRP was visualized using the HIGHDEF blue chromogen substrate (Enzo Life Sciences, Farmingdale, NY, USA). A mixture of pre-immune mouse sera (n = 3, 1:200 dilution) was used as a negative control. The slides were covered with Permount and observed under a light microscope. The rat livers infected with *C*. *sinensis* were similarly examined with the mouse antisera and the HIGHDEF red chromogen.

## Results

### Identification of *C*. *sinensis* globin sequences

BLAST searches of the amino acid sequences of human globins identified six paralogous proteins in the GenBank proteomic database of *C*. *sinensis*, which were annotated as myoglobin (GAA56719/AAM18464/AAN28366, 100/150/150 aa, and GAA32735, 149 aa), ataxia telangiectasia mutated family protein (GAA56508, 146 aa), neuroglobin (GAA51754, 274 aa), globin X (GAA47520, 303 aa), and flavohemoglobin (GAA58066, 352 aa). The protein assigned to GAA56719 was likely an artificially truncated version of the AAM18464/AAN28366 protein considering their sequences and the molecular weight of the corresponding native myoglobin [32]. The *C*. *sinensis* globins were separately clustered in a guide tree together with their respective orthologs identified in the nematodes and trematodes as well as human and zebrafish (S1 Fig). These proteins were named *C*. *sinensis* myoglobin 1 (CsMb1, AAM18464), CsMb2 (GAA32735), CsMb3 (GAA56508), neuroglobin (CsNgb), globin X (CsGbX), and flavohemoglobin (CsFHb) in this study.

### Structural characterization

**Fig 1:**
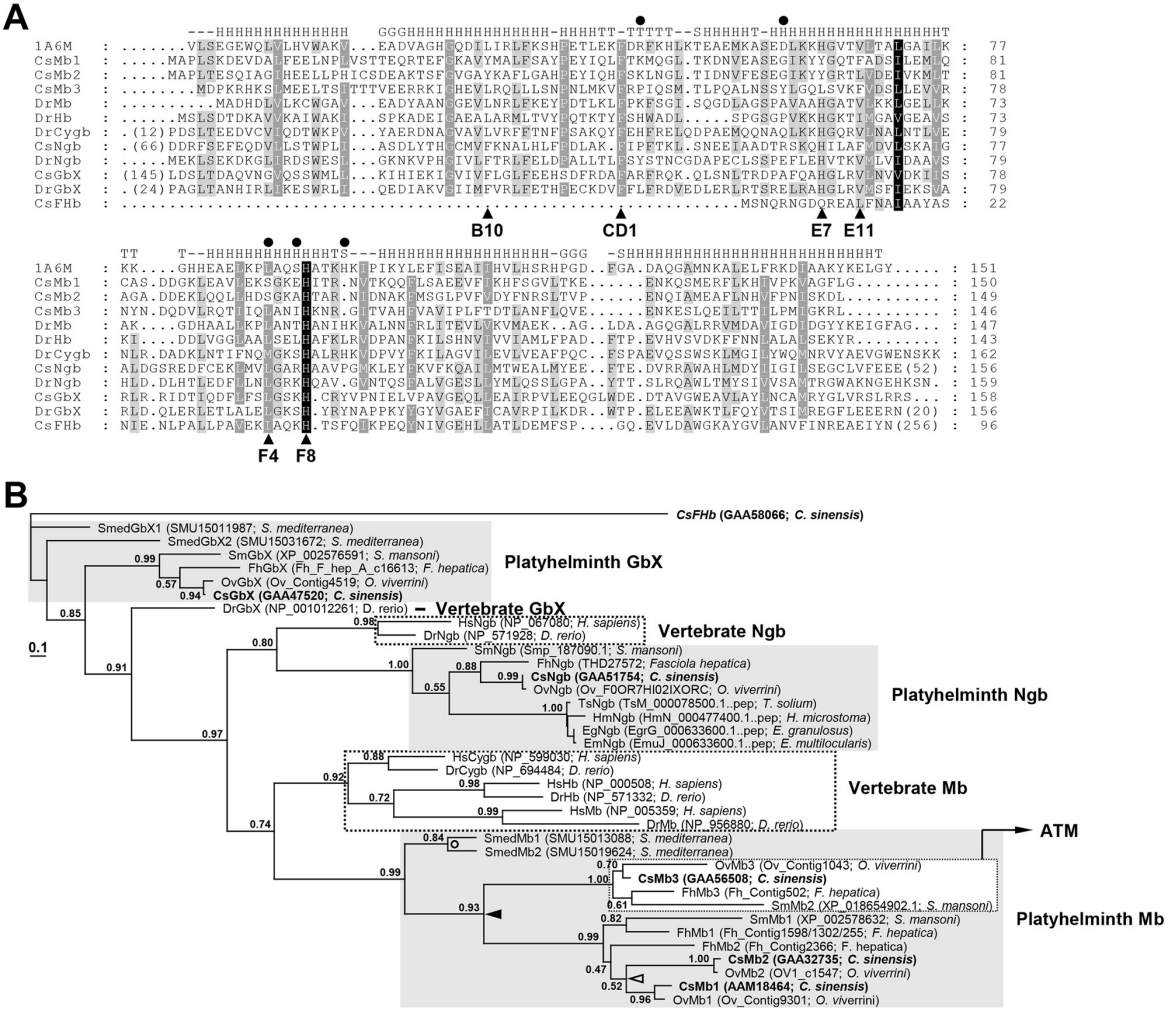
Structural features of the *Clonorchis sinensis* globins. **A.** The amino acid sequences of the *C*. *sinensis* proteins were aligned with those of *Physeter catodon*, of which the tertiary structure was empirically determined (PDB no. 1A6M), and *Danio rerio* orthologs. The degree of amino acid conservations was highlighted by different shades of grey, and gaps were introduced in the alignment to increase the similarity values. The amino acid positions involved in the binding of heme and oxygen molecules are marked with solid arrowheads and circles (see text). Secondary structure elements determined in the *P*. *catodon* globin are shown on the top of the alignment (-, no secondary structure assigned; S, bend; T, turn; G, 3/10-helix; H, alpha-helix). **B.** A phylogenetic tree of *C*. *sinensis* globins and their orthologs. The maximum likelihood tree was constructed using PhyML based on the sequence alignment of globin domains and was rooted in CsFbH. *C*. *sinensis* proteins are highlighted in boldface. Branch support values > 0.50, which were obtained by the Shimodaira-Hasegawa-like approximate likelihood ratio test, are indicated at the corresponding branching nodes. The solid arrowhead indicates an evolutionary point where the progenitor of the platyhelminth Mb gene might have been duplicated.

The *C*. *sinensis* globin sequences were compared with those of the sperm whale (*Physeter catodon*, PDB: 1A6M), the tertiary structure and key amino acid positions of which were well characterized [33], and zebrafish globins (Fig 1A). These proteins well preserved the proximal His, which forms a covalent bond with a heme-coordinated iron atom to stabilize the heme-Fe-myoglobin complex, at a position corresponding to F8 of the sperm whale myoglobin. However, the distal His occupying the E7 helical position to form a hydrogen bond with an oxygen molecule in oxygenated myoglobin was replaced with either Tyr in CsMb1 and CsMb2 or Leu in CsMb3. The other amino acids linked to the propionate groups of heme via hydrogen bonds were also variously substituted in the *C*. *sinensis* proteins (solid circles in Fig 1A). The non-polar amino acid Leu or Phe was replaced with Tyr at B10, which can provide an additional hydrogen bond between myoglobin and bound oxygen [13,34]. Meanwhile, amino acids occupying other invariant sites such as CD1, where the aromatic ring makes contact with the heme, and E11, which forces the oxygen molecule to tilt away from a preferred perpendicular alignment with the plane of the heme, were relatively well conserved in these globin proteins (Fig 1A).

### Phylogeny of *C*. *sinensis* globins

The guide tree of platyhelminth and nematode globins constructed by Clustal X demonstrated that the genomic dosage of the globin genes expanded/shrunken uniquely during the divergence of platyhelminth and nematode species (S1 Fig). For a more detailed investigation of globin evolution in platyhelminths, globin sequences were further isolated from the proteomic/genomic databases of trematodes and cestodes as well as a turbellarian *S*. *mediterranea*, of which draft whole-genome sequence information was publicly available. The isolated sequences were aligned with those of the human and zebrafish globins for a phylogenetic analysis.

**Fig 2:**
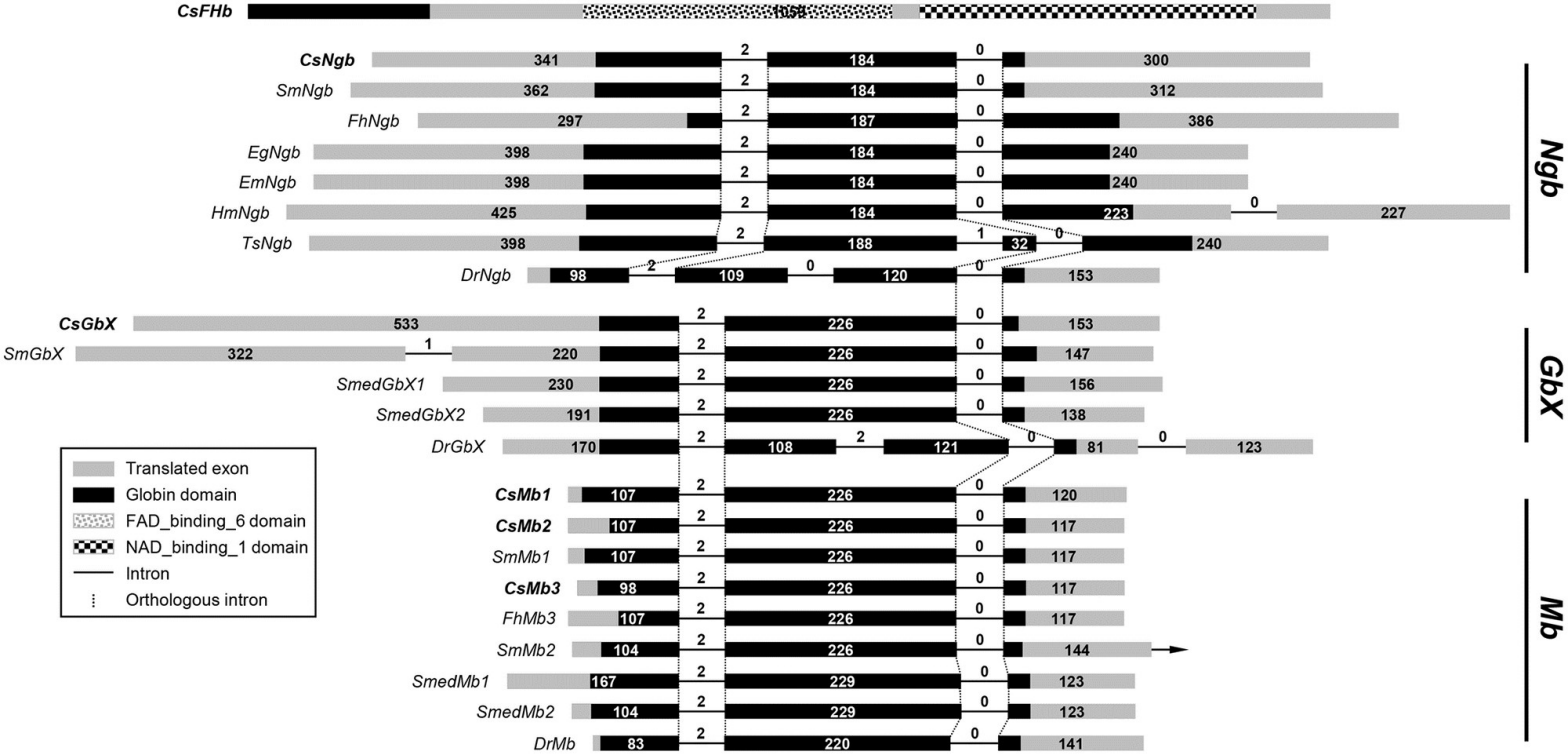
The exon-intron organizations of the *Clonorchis sinensis* globin genes. Genomic organizations of the *C*. *sinensis* genes and their orthologs were determined by comparing the genomic and transcribed sequences of the respective genes. The coding DNA sequences (CDSs) are represented with gray squares in proportion to their relative sizes and the intervening introns are indicated by solid lines with a fixed length. The phase of each intron, as well as the length of exons and introns in base pairs, is indicated in parentheses. Introns occupying orthologous positions among these genes are connected by vertical dotted lines. The positions of codons encoding functional domains, which are listed in the legend, are differentially marked in the respective CDS regions.

The globin family members exhibited unequal distributions across the platyhelminth taxa. The trematode species possessed proteins showing greater structural affinities toward each of the GbX, Ngb, and Mb subfamilies, whereas the cestode species had a single protein similar to Ngb. The GbX- and Mb-like proteins, but not Ngb-like protein, were also detected in the turbellarian. Unexpectedly, the CsFHb protein that showed a structural similarity pattern biased toward bacterial FHbs did not retrieve any ortholog from these platyhelminth species. In a maximum-likelihood tree rooted with CsFHb, the major *C*. *sinensis* proteins were separately positioned in platyhelminth Ngb (SH-aLRT value, 1,00), GbX, and Mb (SH-aLRT value, 0.99) clades, together with their corresponding orthologs (Fig 1B). The platyhelminth Mb clade formed a monophyletic group with a vertebrate clade containing Mb, Cygb, and Hb (SH-aLRT value, 0.74), which is known to be produced by vertebrate-specific duplication events [17]. The ancestral Mb gene was also specifically duplicated in the progenitor of trematodes after the divergence of Turbellaria (closed arrowhead in Fig 1B). One of the daughter copies was additionally duplicated in the common ancestor of *C*. *sinensis* and *O*. *viverrini* (open arrowhead). The ancestral globin gene was duplicated independently in the turbellarian genome (open circle). The phyletic relationships among the globin family members indicated in Fig 1B were supported by the presence of orthologous introns that were commonly or differentially shared in their corresponding chromosomal genes (vertical dotted lines in Fig 2).

### Expression profiles of globin genes related to the *C*. *sinensis* maturation

**Fig 3:**
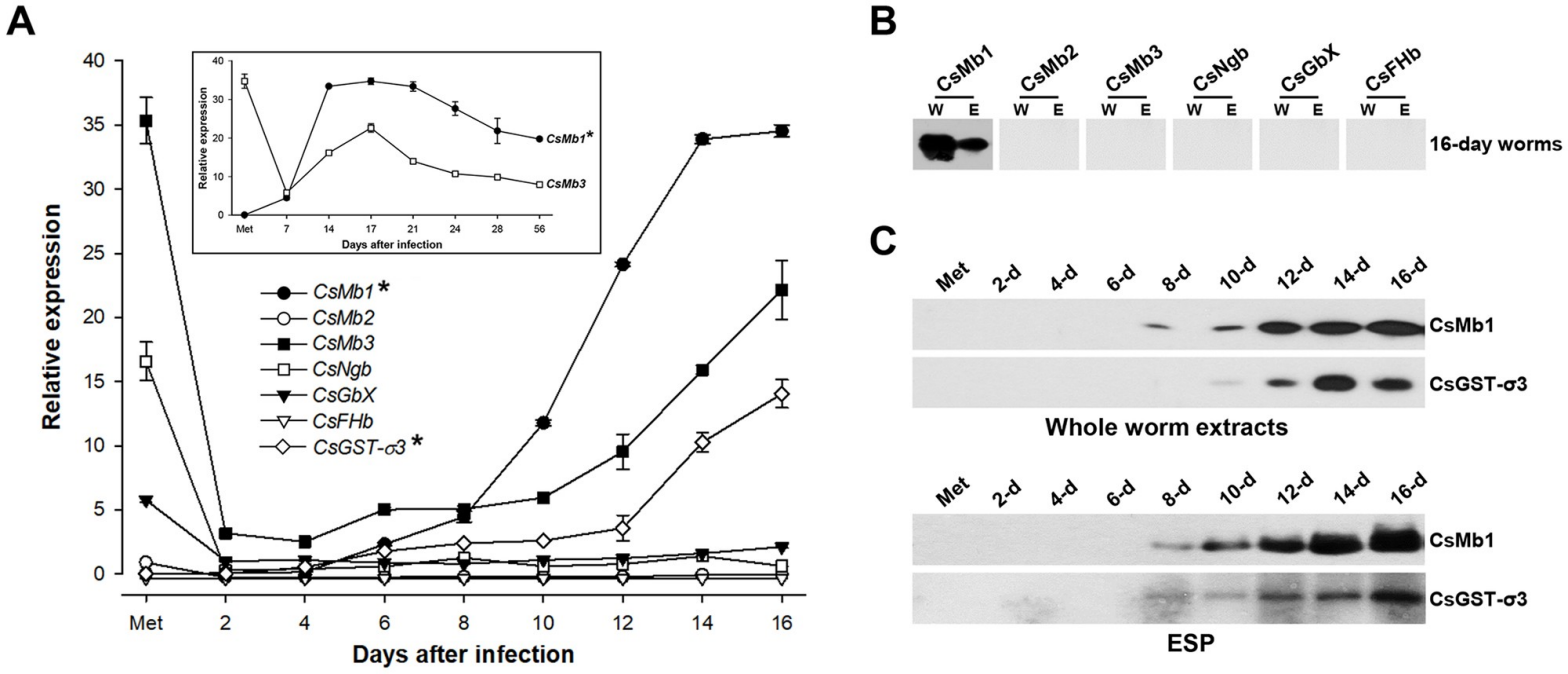
Temporal expression profiles of *Clonorchis sinensis* globin genes. **A.** Induction profiles of the *Clonorchis sinensis* globin genes were assessed using a quantitative PCR (qPCR) method related to the development/maturation of the liver fluke from metacercariae to 16-day worms. The calculations are based on independent technical triplicates (n = 3, mean ± S.D.). In cases of the *CsMb1* and *CsMb3* genes, worms up to 56 days-old were included in the measurements (inset graph). The relative expressions of *CsMb1* and *CsGST-σ3* (marked by asterisks) are given in 1/1,000 of their original values in the graphs. **B**. Western blot analysis of the whole worm extracts (W, 30 μg/well) of *C*. *sinensis* adults and their excretory-secretory products [ESPs (E), 10 μg/well] with antibodies specific to *C*. *sinensis* globins. **C.** The temporal secretion profile of CsMb1 was similarly examined in the whole body extracts and the ESPs of *C*. *sinensis* worms from metacercaria to 16 days post-infection. The anti-CsGST-σ3 antibody was also included in the blotting analyses.

The transcriptional ontogenies of globin genes were assessed in *C*. *sinensis* worms at various developmental and maturation stages ranging from metacercaria to 16-day worm stages (Fig 3A). The relative transcriptional activities of these genes were substantially different at all the stages examined. The order of mRNA transcript levels changed in association with the development from metacercariae (*CsMb3* ≥ *CsMb1*> *CsNgb* > *CsGbX* > *CsMb2)* to 2-day juvenile worms (*CsMb1* >>> *CsMb3* > *CsGbX* > *CsNgb* > *CsMb2*) and then remained the same throughout maturation of the worm. The *CsMb1* transcription level increased linearly in 2-day (3.1-fold) and 4-day (2.1-fold) worms compared to those in previous-stage worms (*P* < 0.01), whereas it began to increase exponentially from 6-day worms by up to three orders of magnitude in 16-day worms. *CsMb3* expression in the 2-day worms was 10-fold lower than that in the metacercariae. The expression level became progressively higher in association with worm maturation (63% recovery in 16-day worms). An investigation of the longer-lived worms up to 56 days demonstrated that the transcriptional activities of *CsMb1* and *CsMb3* reach their maxima in worms aged around 16 days (inset graph of Fig 3A). The transcription levels of *CsMb2*, *CsNgb*, and *CsGbX* also decreased significantly in the 2-day worms (6.4%, 4.0%, and 22.5%, respectively). The decreased expression levels of these genes were maintained during the subsequent parasitic maturation. Meanwhile, the *CsFHb* transcript was rarely detected by qPCR throughout the life stages examined.

The high level of *CsMb1* expression was supported by western blot analyses of whole-worm extracts (30 μg/well) using the recombinant CsMb1-specific antibody (Fig 3B and 3C). Protein extracts from worms over 8 days old, but not those from worms under 6 days, strongly reacted with the antibody in proportion to the maturation of the worm (Fig 3C). None of the blots responded to antibodies specific to *C*. *sinensis* globins other than CsMb1. These negative results might be due to the low expression levels of the corresponding globins below the detection threshold in the analyses (Fig 3A). Western blot analysis of ESP samples (10 μg) further demonstrated that a considerable amount of CsMb1 was secreted into the surrounding milieu from the 8-day worms (Fig 3C). Interestingly, the temporal expression and secretion profiles of CsGST-σ3, which was the second most abundant protein in the *C*. *sinensis* adult ESP [32], were similar to those of CsMb1 (Fig 3A and 3C).

### Effect of oxygen, nitrogen compounds, and bile on the globin gene expression

**Fig 4:**
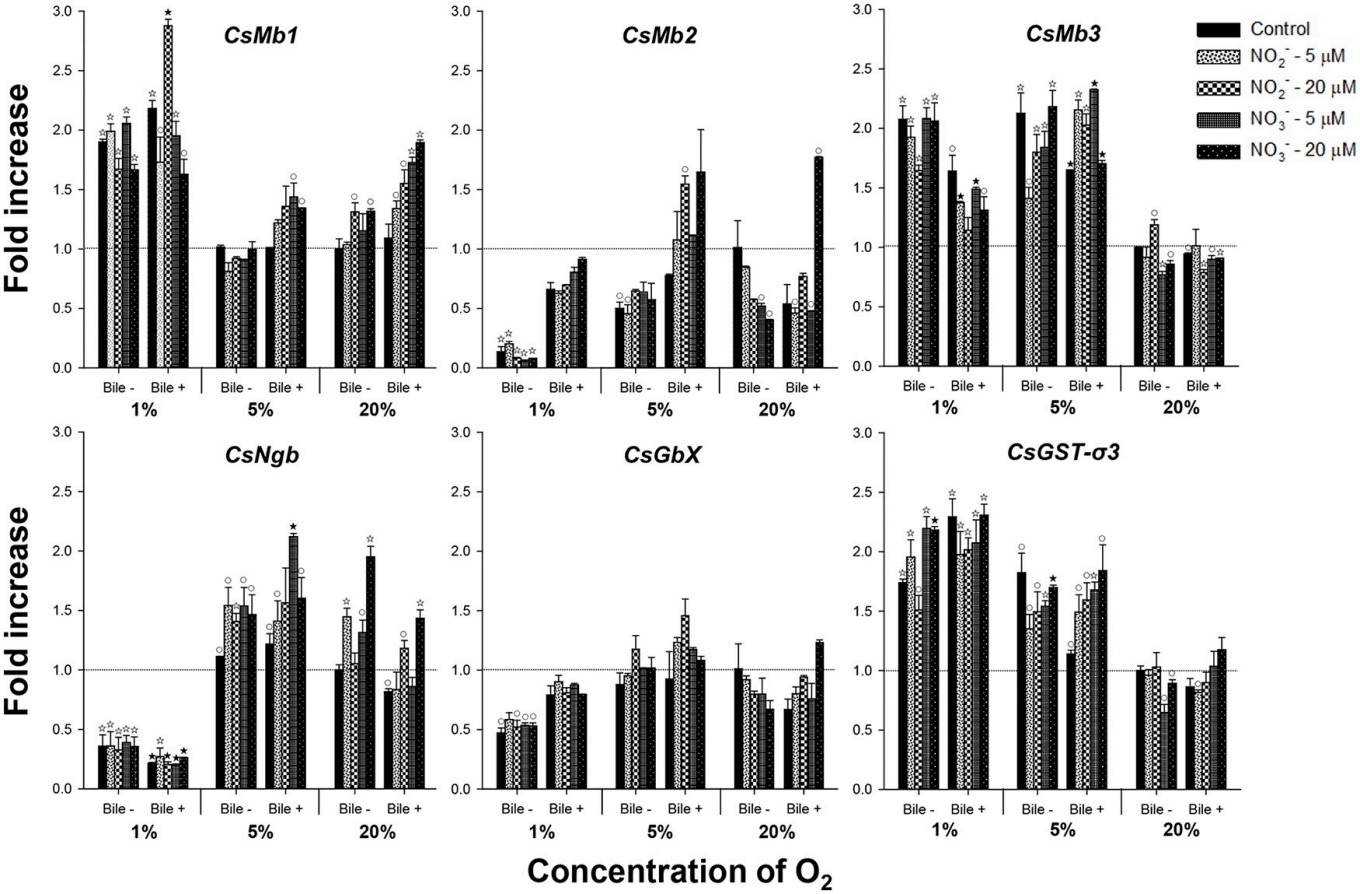
Expression profiles of globin genes in *Clonorchis sinensis* adults incubated under various experimental conditions. Live *C*. *sinensis* worms were incubated in the RPMI-1640 medium supplemented with bile, nitrite, or nitrate under 1%, 5%, or 20% oxygen conditions. After 24 h incubations, mRNA extracted from the worms was used in qRT-PCR with primer sets specific to each of the *C*. *sinensis* globin genes. Fold increases in the globin gene expression in each of the experimental groups were calculated against the 20% oxygen group without any chemical supplement (n = 2, mean ± SD). ^○^*P* < 0.05; ^☆^*P* < 0.01; ^★^*P* < 0.001.

The effect of oxygen on the expression of *C*. *sinensis* globin genes was examined by incubating *C*. *sinensis* worms for 24 h under 1%, 5%, and 20% oxygen conditions. As shown in Fig 4, the *CsMb1* and *CsMb3* expression levels increased significantly by 1.9- and 2.1-folds (*P* < 0.01), respectively, in worms incubated under 1% oxygen condition compared to those in the unincubated control or 20% oxygen groups. Conversely, the levels of *CsMb2* (0.1-fold), *CsNgb* (0.4-fold), and *CsGbX* (0.5-fold) transcription were decreased by hypoxic stimulation (*P* < 0.01). Under the 5% oxygen condition, *CsMb2* and *CsMb3* were downregulated and upregulated (0.5- and 2.1-folds, respectively; *P* < 0.05), but not the other globin genes, compared to their transcription levels under 20% oxygen condition. Nitrite, nitrate, and bile, which were added to the incubation medium, marginally affected globin gene expression depending on the surrounding oxygen concentration, although no clear tendency was observed for their modes of action (Fig 4). *CsGST-σ3* expression was also variably induced by these exogenous stimuli, especially under lower oxygen conditions.

### NO and globin gene expression

**Fig 5:**
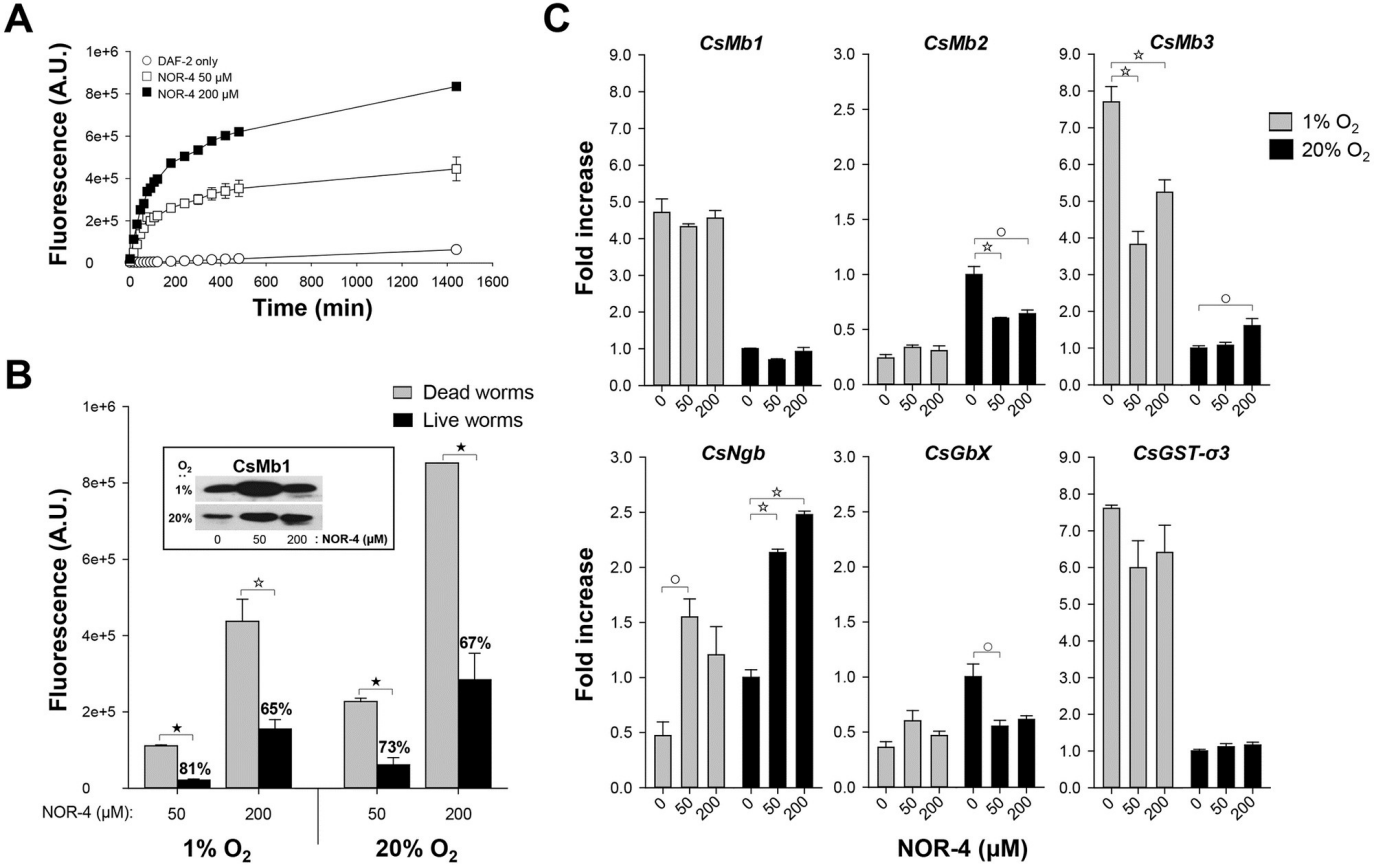
Effect of nitric oxide (NO) on the expressions of the *Clonorchis sinensis* globin genes. **A.** The relative amounts of NO released from NOR-4 in the RPMI-1640 medium was measured using the DAF-2 fluorescence assay method in relation to the incubation times. A.U., arbitrary unit. **B**. Live or dead *C*. *sinensis* adults were incubated for 24 h in the RIMI-1640 medium supplemented with NOR-4 and DAF-2 under 1% or 20% oxygen conditions. The fluorescence values of the conditioned media, which were measured using the DAF-2 method, were serially subtracted from those of NOR-4- and DAF-2-only media (n = 3, mean ± SD). The percentages on the histograms of the live-worm groups indicate the degree of fluorescence reduction compared to those in the dead-worm groups. ^☆^*P* < 0.01; ^★^*P* < 0.001. The results of western blot analysis of the conditioned media (20 μL/well) with an anti-CsMb1 antibody are shown in the inset image. **C**. The expression levels of the globin genes were examined in the *C*. *sinensis* worms described in panel B by performing qRT-PCR. The fold increases were calculated by comparing the values of the experimental groups with those in the 20% worm-only control group (n = 3, mean ± SD). ^○^*P* < 0.05; ^☆^*P* < 0.01.

The generation of NO by NOR-4 in the RPMI-1640 medium increased constantly during the first 4 h in proportion to the NOR-4 concentration and then gradually reached a plateau (Fig 5A). When *C*. *sinensis* worms were incubated in the NOR-4-containing medium for 24 h under 1% and 20% oxygen conditions, the fluorescence units were also elevated depending on the NOR-4 and oxygen concentrations. However, the values of the live worm-conditioned media were significantly lower than those of the respective controls with dead worms (*P* < 0.01; Fig 5B). The percentage decrease was more prominent in the media with 50 μM NOR-4 than in media containing 200 μM NOR-4 (81% vs. 65% and 73% vs. 67% under the 1% and 20% oxygen conditions, respectively; *P* < 0.01). Western blot analysis of these conditioned media with the CsMb1-specific antibody demonstrated that CsMb1 secretion was strongly induced by the addition of NOR-4, although the secretion was suppressed in the media with 200 μM NOR-4 under the 1% oxygen condition (inset of Fig 5B). Meanwhile, transcription of these *C*. *sinensis* genes was not drastically affected by exogenous NO, except for *CsNgb*, which was significantly activated by the chemical (*P* < 0.05; Fig 5C).

### Co-incubation of *C*. *sinensis* adults and human cholangiocytes

**Fig 6:**
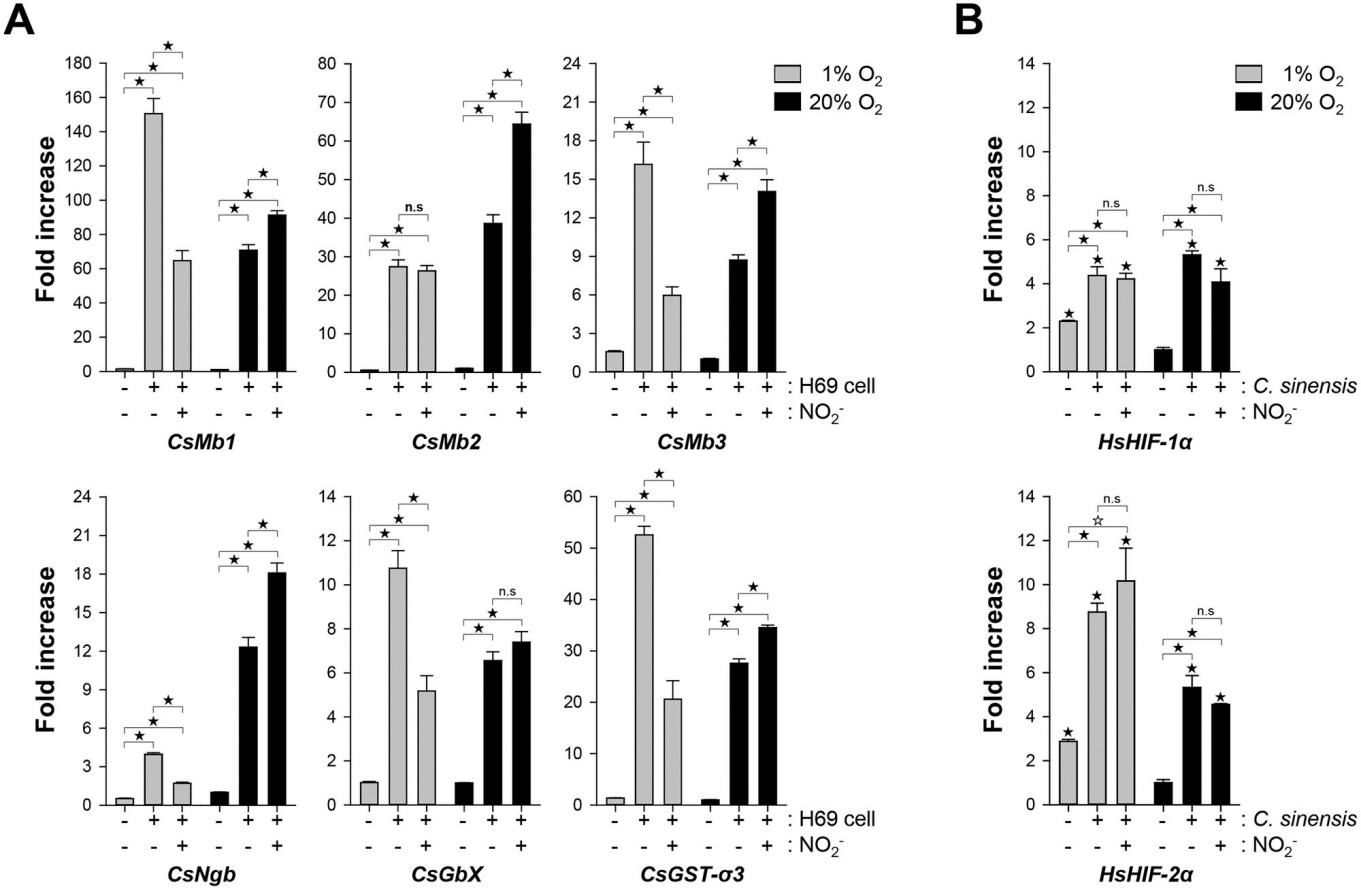
Induction profiles of globin and hypoxia-inducible factor (HIF) genes in co-incubated *Clonorchis sinensis* adults and H69 cells. **A**. The live 28-day-old worms were co-incubated with the human cholangiocytes under the respective conditions. After 24 h incubation, the relative amounts of the *C*. *sinensis* gene transcripts were determined using a qRT-PCR method (n = 3, mean ± SD) and the fold increase in each of the experimental groups was calculated against that in the worm-only control group under 20% oxygen condition. **B**. Fold increase in the expression of *HIF-1α* and *2α* was similarly determined in the human cells. ^☆^*P* < 0.01;^★^*P* < 0.001; n.s., not significant.

The 28-day-old *C*. *sinensis* worms were co-incubated with H69 cells under 1% and 20% oxygen conditions. After 24 h, RNA samples extracted from these worms were used for quantification of the globin gene transcripts using qPCR. As shown in Fig 6A, the transcription level of all these globin genes was greatly induced by co-incubation compared to those in the worm-only control groups [7.58- (*CsNgb*)—106.70- (*CsMb1*) fold increase under 1% oxygen and 6.56- (*CsGbX*)—70.83- (*CsMb1*) fold increase under 20% oxygen; *P* < 0.001]. Interestingly, the expression of these genes was significantly downregulated or upregulated by the addition of nitrite in the co-incubation medium. The fold change ratios of *CsMb1*, *CsMb2*, *CsMb3*, *CsNgb*, and *CsGbX* in the co-incubation group with nitrite to those in the group without nitrite were 0.43, 0.96, 0.37, 0.43, and 0.48, respectively, under the 1% oxygen condition (*P* < 0.001, except for *CsMb2*). The respective ratios increased to 1.29, 1.67, 1.61, 1.47, and 1.13 (*P* < 0.001, except for *CsGbX*) under the 20% oxygen condition. *CsGST-σ3* showed induction patterns under the experimental conditions similar to, but less extensive than, those of *CsMb1* (Fig 6A). Coinciding with the upregulated responsiveness of *C*. *sinensis* globin genes, the transcriptional activities of human *HIF-1α* and *HIF-2α* were significantly increased in H69 cells (Fig 6B). The effect of nitrite on human gene expression was negligible. Similar fold-change patterns of these gene expressions were observed in an additional batch experiment using 56-day-old worms, even though the levels of respective fold changes were substantially reduced in this replicated study (S3 Fig).

### Histological distributions of *C*. *sinensis* globins

**Fig 7:**
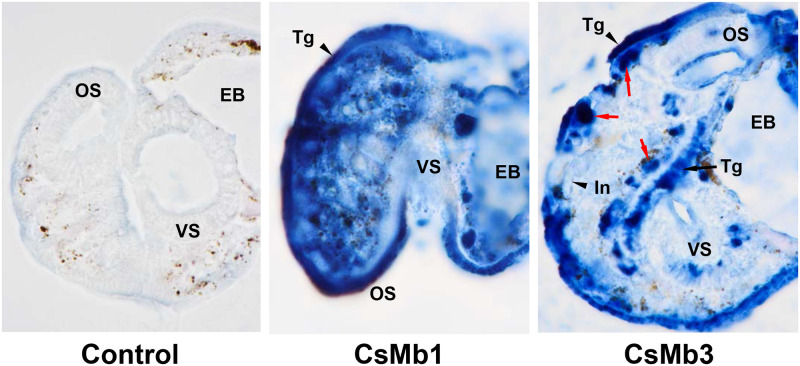
Detection of CsMb1 and CsMb3 in the metacercarial stage of *Clonorchis sinensis*. The whole-body sections of *C*. *sinensis* metacercariae embedded in paraffin were reacted with mouse antibodies specific to the recombinant forms of *C*. *sinensis* CsMb1 and CsMb3. Pooled serum from pre-immune mice (n = 3) was used in the control reaction. The positive reactions were developed into a blue color using a blue chromogen substrate for horseradish peroxidase-conjugated to a second antibody. The red arrows mark a follicle-like structure exhibiting a strong positive signal against the anti-CsMb3 antibody in the subtegumental region. EB, excretory bladder; In, intestine; OS, oral sucker; Tg, tegument; VS, ventral sucker. Original magnifications, x 1,000.

**Fig 8:**
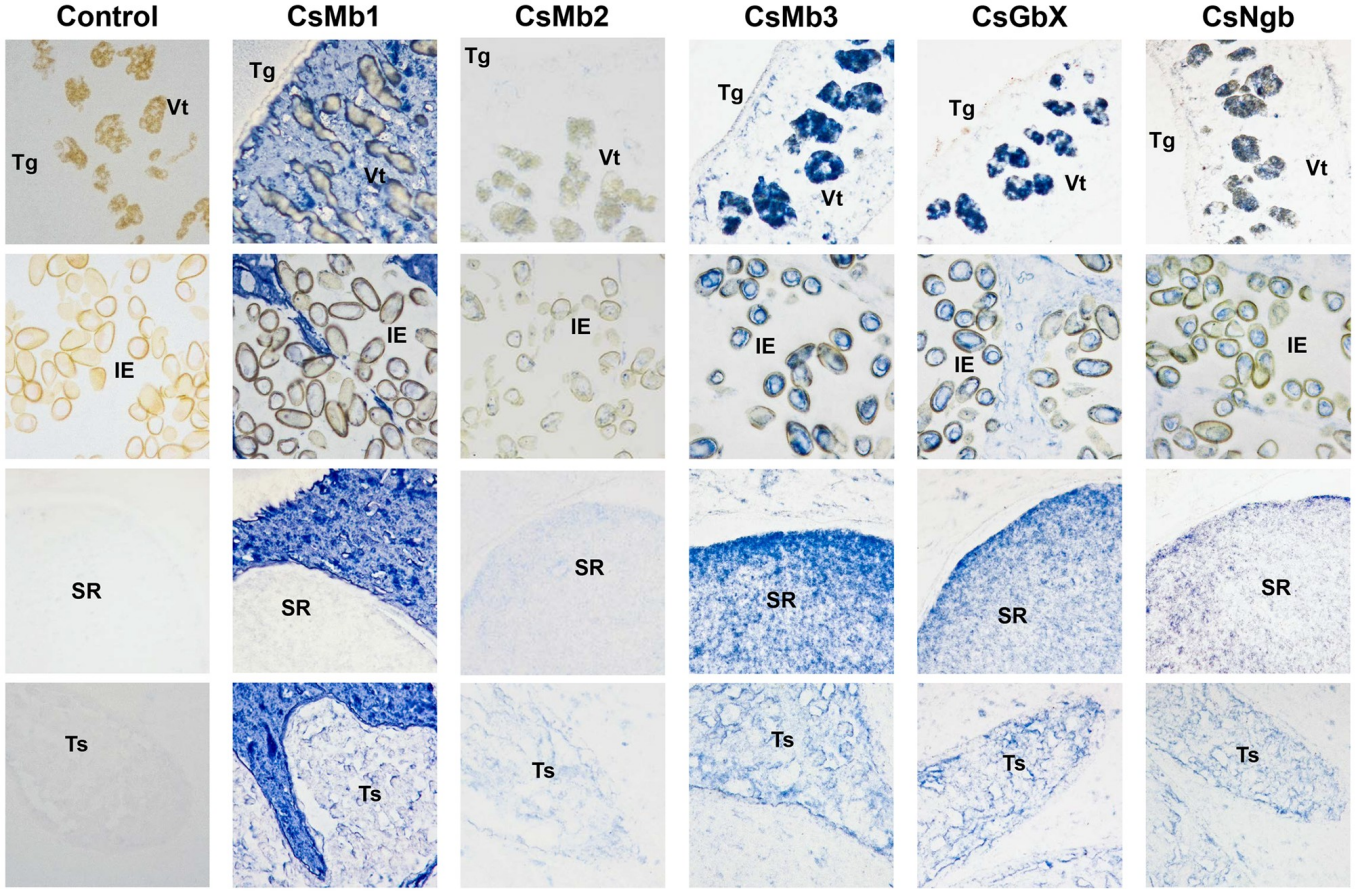
Histological distribution patterns of globins in *Clonorchis sinensis* adults. The worm sections were incubated with respective mouse antibodies specific to the *C*. *sinensis* globins or a pooled pre-immune mouse serum (n = 3) and then a goat anti-mouse IgG antibody conjugated with horseradish peroxidase (HRP). The specific antigen-antibody reactions were developed into a blue color with a blue chromogen for HRP. IE, intrauterine eggs; SR, seminal receptacle; Tg, tegument; Ts, testis; Vt, vitellaria. Original magnifications, x 200.

Histological distribution of globins was assessed in whole-body sections of *C*. *sinensis* metacercariae and adults using mouse antisera specific to the corresponding recombinant proteins. In metacercaria, the CsMb1-positive signal was distributed widely throughout the parenchyma and tegument, whereas that against CsMb3 was mainly detected in the follicular forms in the subtegumental region between the oral and ventral suckers (red arrows) as well as in the tegument (Fig 7). Antibodies specific to the other *C*. *sinensis* globins did not show any considerable signal, presumably owing to their low expression levels in metacercaria (Fig 3A). The histological distribution patterns of globins were dichotomized between the parenchymal region and sexual organs in adult worms. The major staining signal with anti-CsMb1 antibody was observed in the parenchymal region, whereas those with the other globin-specific antibodies were in the sexual organs such as vitellaria, seminal receptacle, testes, ovaries, and vas deferens. A positive signal was detected in the intrauterine eggs in reactions with the anti-CsMb3, CsGbX, and CsNgb antibodies but not with the anti-CsMb1 antibody (Fig 8). The antibody against CsMb2, the gene for which showed a minimal expression level (Fig 3), exhibited a staining pattern similar to, but much weaker than that of CsMb3 (Fig 8). None of the *C*. *sinensis* globins was detected in the tegument of adult worms (Fig 8).

**Fig 9:**
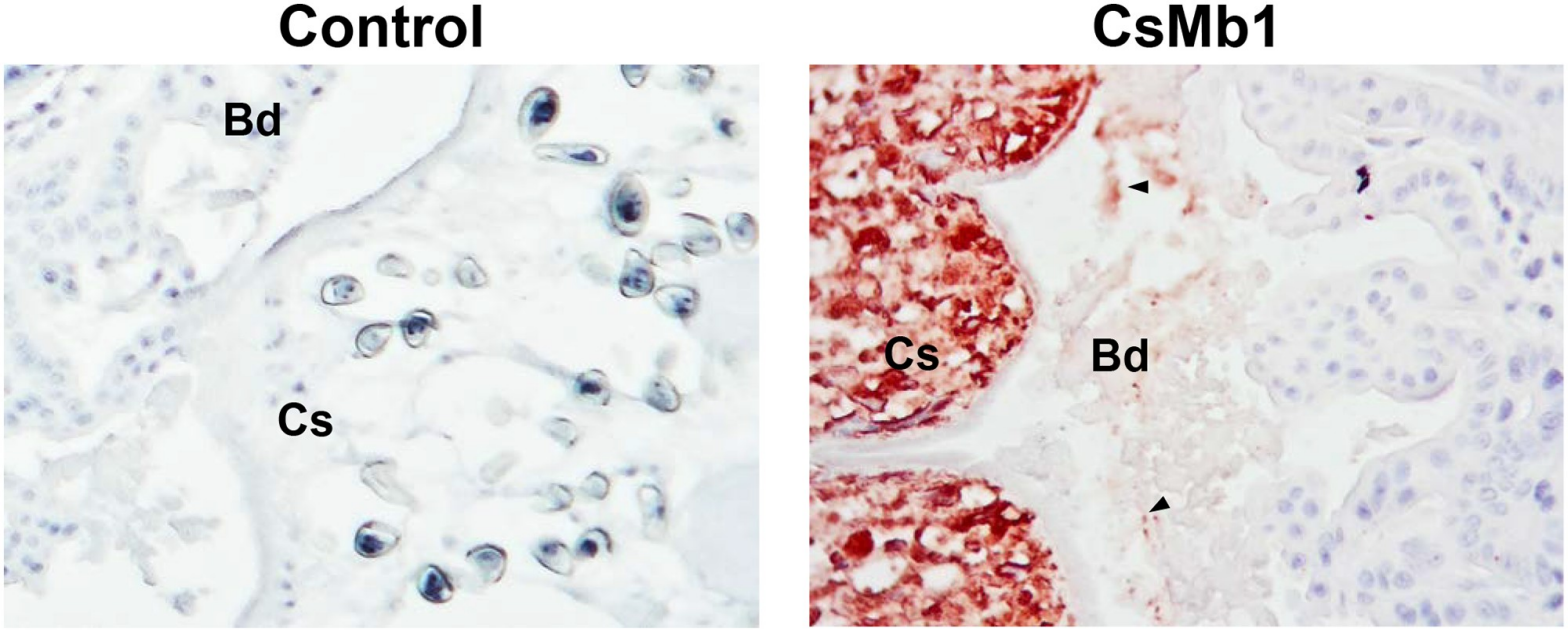
Detection of secreted CsMb1 in the epithelium of rat bile ducts with clonorchiasis. Histologic sections containing the intrahepatic bile ducts, which were prepared from the liver of an experimental rat with clonorchiasis, sequentially reacted with an anti-CsMb1 antibody or pooled mouse serum (n = 3) and a goat anti-mouse IgG antibody conjugated with horseradish peroxidase (HRP). The positive reactions were visualized by staining with a red chromogen substrate for HRP. The arrowheads indicate examples of the strong positive signal. Bd, epithelium of the bile duct; Cs, *Clonorchis sinensis* worm body. Original magnifications, x 200.

The epithelial barrier of the bile ducts near the infection sites was heavily corrupted in the experimental rat models of clonorchiasis. Furthermore, *in situ* immunohistochemical staining of the rat livers with the CsMb1-specific antibody showed that the secreted CsMb1 molecules gained access to the liver parenchyma through the damaged epithelia (arrowheads in Fig 9).

## Discussion

In the present study, the structural and expressional properties of six globin genes and proteins were investigated in *C*. *sinensis*. The globin domains conserved in the primary structures of these proteins exhibited much closer similarities to members of the Mb (CsMb1—CsMb3), Ngb (CsNgb), GbX (CsGbX), and FbH (CsFbH) subfamilies, respectively. Expression of the *Clonorchis* globin genes increased exponentially in the worms coinciding with their sexual maturation, after being downregulated in the juveniles compared to those in the metacercariae. The CsMb1 protein was strongly detected throughout the parenchymal region of the adult worms as well as in the ESP, whereas the other proteins were localized exclusively in the sexual organs and intrauterine eggs. External stimuli, including oxygen, NO, and nitrite, variously affected globin gene expression in adult worms, which may suggest functional diversification among them. These genes were also highly inducible in *C*. *sinensis* worms incubated with human cholangiocytes.

In vertebrates, multiple *Mb* subfamily genes (*Mb*, *Hb*, and *Cygb*) are generated by a series of gene duplications, followed by functional diversification, ranging from the maintenance of oxygen supply to the regulation of redox homeostasis. The *Ngb* gene product seems to be involved in redox-regulated signaling and oxygen-sensing processes in the nervous system and some endocrine tissues of vertebrates. The *GbX* gene product plays an antioxidant role within a narrow range of donor organisms, including teleost fishes, amphibians, and reptiles [17]. The trematode genomes possessed several genes homologous to the *Mb*, *Ngb*, or *GbX* subfamilies. However, the cestode genomes contained only a single *Ngb*-like gene (Fig 1B). Together with the differential responsiveness of *C*. *sinensis* globin genes to hypoxic conditions (Fig 4), these data suggest that the globin gene has also been subject to lineage-specific amplification/deletion and subsequent functional diversification events in the Platyhelminthes, similar to, but to a lesser extent than in vertebrates [17] and nematodes [20]. Meanwhile, *FHb* orthologs were identified only in *C*. *sinensis* and *C*. *remanei* among the trematodes and nematodes examined in this study (S1 Fig). The genomic scaffold sequence (BADR02005738, 1,092 bp) containing the intronless *CsFHb* gene (1,059 bp) was identical to the chromosomal region sequence of *E*. *coli*, and no ortholog was detected in the *O*. *viverrini* genome. Therefore, the *CsFHb* gene, as well as the *C*. *remanei FHb* gene, seemed to have originated from the chromosomal DNA of the common enteric bacteria contaminating the helminth materials during experimental preparation.

Proteomic detection of parenchymal and/or secreted Mb in adult liver flukes [32,35–37] is highly provocative because these flatworms are believed not to perform aerobic respiration in the hypoxic bile ducts of their mammalian hosts [7,38]. Based on their biochemical properties, these trematode Mbs have been shown to participate in oxygen scavenging, antioxidant defense, and NO deoxygenation, instead of the conventional oxygen transport/storage function [13,34,39]. However, recent findings on the abundant expression of oxidative phosphorylation-related genes in the opisthorchiids as well as in *F*. *hepatica* [9,10,40,41] raise the possibility that trematode Mbs perform physiological role(s) associated with oxygen uptake. Red blood cells in the liver sinusoids supply oxygen to the hepatocytes during their flow from the portal vein/hepatic artery to the central vein [42]. The local oxygen concentration is highest in the periportal region proximal to the portal triad, which constitutes the portal vein, hepatic artery, and bile duct [43,44]. Trematode Mbs exhibit oxygen affinities higher than those of mammalian Hbs, mainly due to Tyr residues at the amino acid positions of B10 and E7 [13,45]. Of the multiple *C*. *sinensis* globins, only CsMb1 with Tyr at these positions was highly secreted into the surrounding host tissue (Figs 3 and 9) and the expression/secretion levels were greatly increased under hypoxic conditions or by co-incubation with human H69 cells (Figs 4 and 6). Furthermore, the CsMb1 expression profile coincided well with that of CsHIF-1α [46]. Taken together, these observations seem to provide a strong rationale for the suggestion that CsMb1 is involved in the active transport of oxygen from the periportal region to the *C*. *sinensis* worm body, similar to mammalian Mb acting in peripheral tissues with oxygen affinity higher than that of hemoglobin [47].

*F*. *hepatica* generates ATP via both aerobic and anaerobic respiration in the livers of mammalian hosts. The ratio of aerobic to anaerobic metabolism drastically decreased after the initiation of sexual reproduction in the bile ducts, even though the capacity of the worm to perform aerobic respiration continuously increased in proportion to the maturation of the worm [40]. The aerobic respiratory rate is also very low in the female body of *S*. *mansoni* parasitizing oxygen-rich blood vessels, which is in contrast to the situation in its male counterpart [48]. In the experimental rats, the expression/secretion of CsMb1 closely coincided with sexual maturation of *C*. *sinensis* (Fig 3; [12]). Moreover, the redox challenge generated by aerobic respiration seemed to be minimal in the parenchymal region of *C*. *sinensis* [28,49]. Therefore, the oxygen molecules transported into the bodies of the fluke via the action of the secreted Mbs are likely to be consumed most, if not entirely, in a non-respiratory biological process in these adult hermaphroditic or female worms. The tanning process responsible for the generation of sclerotin-type eggshells is the major oxygen-consuming metabolic pathway in trematode species [7]. In this context, it is plausible that the genes encoding the Mb subfamily have been deleted in the cyclophyllidean cestode genomes following metabolic changes in the eggshell hardening, or vice versa (Fig 1B). Unlike trematodes and pseudophyllidean cestodes generating the sclerotin-type eggshell, the cyclophyllidean cestodes produce eggs surrounded by a thin keratin-type eggshell [50]. The tyrosinase gene, of which the product mediates the tanning process by exhausting oxygen, was also not identified in the genomes of the cyclophyllidean cestodes [51].

The egg-laying capacity of *C*. *sinensis* adults rapidly increases during the early stage of infection and then gradually decreases with time after reaching a peak in experimental animals [52]. The reproductive period and fecundity are dependent on several factors, such as host species and infection rate [53]. The ontogenic expression patterns of *CsMb1* and *CsMb3* (Fig 3A), as well as those of tyrosinase genes [51], might be closely related to the age-specific fertility rate of *C*. *sinensis* worms in the experimental host. It could also be suggested that the differential induction levels of globin genes against H69 cell-induced hypoxia, which were observed between 28-day- and 56-day-old worms (Fig 6 and S3 Fig), are due to differences in the oxygen demands largely related to the fecundity of the worms.

In addition to the functions related to oxygen uptake, Mb homologs are known to exhibit extra enzymatic activities, including NO dioxygenase (oxy-form) and nitrite reductase (deoxy-form) depending on their oxygenation status [54]. NO and nitrite influenced the *CsMb1* gene expression at the protein and mRNA levels, respectively, in *C*. *sinensis* worms (Figs 5B and 6A), although the transcript level was not significantly altered in worms treated with NO (Fig 5C). The discrepancy in protein and its coding transcript levels may demonstrate that any kind of post-transcriptional and post-translational mechanism, which modulates translation rate of mRNA, half-life of the translated protein, or spatial location of the protein, contributes to the establishment of the CsMb1 expression level for the short-term temporal adaptation [55]. It is also possible that the change in the concentration of the *CsMb1* transcript, if any, was not distinguishable due to a technical error associated with preparing RNA samples. In this study, the probable technical error could not be statistically estimated, since a pooled RNA sample from each of the experimental groups was used in qPCR. Nonetheless, these data strongly suggested that the secreted CsMb1 mediates an additional biochemical pathway(s) other than oxygen uptake in host environments through these enzymatic activities. Several liver cells, such as hepatocytes, cholangiocytes, stellate cells, and Kupffer cells, generate NO via the action of inducible NO synthase under inflammatory conditions, including *C*. *sinensis* infection [56,57]. The relative levels of NO decreased significantly in the NOR-4-containing media when incubated with live *C*. *sinensis* worms, which was accompanied by increases in CsMb1 secretion and *CsHIF-1α* expression (Fig 5; [46]). Therefore, it is likely that a significant fraction of the oxygenated CsMb1 participates in the conversion of cytopathic NO into nitrate, thereby resulting in hypoxia within the bodies of the worms. Under hypoxic conditions with nitrite, the deoxygenated CsMb1 changes to an oxygenated form by virtue of its nitrite reductase activity, which in turn downregulates the expression of *CsMb1* and *CsHIF-1α* (Fig 6 and S3 Fig; [46]). On the contrary, the *HIF-1α* and *HIF-2α* genes were not significantly affected in H69 cells lacking *Mb* expression in the presence of nitrite. The biochemical reaction for the *de novo* generation of oxygenated CsMb1 can possibly occur in the hypoxic bile ducts, since biliary secretion is one of the major routes to excrete excess nitrite and nitrate [58]. It has been shown that *Mb* gene expression is induced via HIF-1α transactivation in vertebrates [59]. Further studies to address issues regarding the regulatory signals of *CsMb* gene expression in association with *CsHIF-1α* and the enzymology of these gene products are needed to verify the proposed physiological functions of CsMb1 in *C*. *sinensis*. Information on the shuttle pathway of CsMb1 across the *C*. *sinensis* tegument will also be essential to verify its proposed physiological role in oxygen uptake from host environments.

Regardless of the source, host cells or the probable CsMb1-mediated reduction of nitrite, NO can pose a serious threat to the survival of the parasite. NO is readily transformed into S-nitrosoglutathione (GSNO) or further into dinitrosyl-diglutathionyl-iron complex (DNDGIC) by interacting with glutathione (GSH) under *in vivo* conditions. The resulting DNDGIC undergoes time- and concentration-dependent decomposition and acts as a reservoir for the release of NO. However, the natural NO carrier can be greatly stabilized when bound to glutathione transferases (GSTs) [60,61]. Therefore, the GST-mediated stabilization of DNDGIC might alternatively be proposed as an NO detoxification mechanism via sequestration of the cytopathic molecule [61]. It has been reported that *C*. *sinensis* adults secrete multiple GSTs; of these, CsGST-σ3 is the second most abundant protein identified in the *C*. *sinensis* ESP [32]. The correlated gene expression patterns between *CsMb1* and *CsGST-σ3* observed in this study may reflect that the physiological functions of these proteins are closely linked to each other (for example, obtaining oxygen via CsMb1-mediated nitrite reduction and sequestration of consequently generated NO under hypoxic conditions). Further investigations are warranted to reveal the speculated cross-link between CsMb1-mediated oxygen and CsGST-σ3/GSH-mediated NO metabolism.

In conclusion, *C*. *sinensis* expressed multiple Mb homologs that were highly contrasted with other trematode and cestode species. Based on the responsiveness to hypoxia and histological localities, CsMb1 and CsMb3 may serve as canonical oxygen suppliers in the parenchyma and intrauterine eggs of the worms as well as the sex organs for energy metabolism and sexual reproduction, respectively, while the other Mb homologs are involved in non-respiratory functions such as regulation of redox homeostasis. Given the induction profiles under nitrosative stress conditions, the secreted CsMb1 seemed to play an additional role in parasite survival by scavenging NO generated by the host immune cells. It was also apparent that *C*. *sinensis* competed with hepatobiliary cells to use oxygen. Prolonged exposure of cells to a hypoxic environment leads to genetic/genomic instability via the elevated frequency of DNA breaks and accumulation of DNA replication errors. Hypoxia further regulates cellular metabolism and angiogenesis, both of which are intimately related to the survival and dissemination of cancer cells [62]. The genomic instability and/or physiological alterations in cholangiocytes induced by the *C*. *sinensis* infection-derived hypoxia could promote onset and progression of cholangiocarcinoma. Therefore, our study provides a molecular clue to understanding the possible mechanisms of cholangiocarcinogenesis caused by *C*. *sinensis* infection. Even though a large amount of CsMb1 is secreted into the host environment, it does not induce specific humoral immune responses in clonorchiasis patients [63]. Therefore, the protein can be further targeted in studies on the downregulation of humoral immune responses by *C*. *sinensis* and/or the development of protective vaccines against the highly carcinogenic liver fluke.

## Supporting information

S1 Fig A guide tree of globins showing differential multiplication/loss of globin genes among trematode and nematode species. The trematode (red) and nematode (black) sequences were retrieved from the GenBank databases during similarity searches of *Clonorchis sinensis* globins using the BLAST programs. Sequences of human (*Homo sapiens*; Hsap) and zebrafish (*Danio rerio*; Drer) globins (blue) were also targeted in the analyses. The tree was constructed using Clustal X during the alignment of the retrieved sequences. The branches connecting members of each globin subfamily are differentiated from one another by colors identical to those of the corresponding subfamily names. The *C*. *sinensis* proteins are highlighted in boldface. Csin, *C*. *sinensis*; Dden, *Dicrocoelium dendriticum*; Fhep, *Fasciola hepatica*; Ihyp, *Isoparorchis hypselobagri*; Pepi, *Paramphistomum epiclitum*; Pwes, *Paragonimus westermani*; Sjap, *Schistosoma japonicum*; Sman, *Schistosoma mansoni*; Acan, *Angiostrongylus cantonensis*; Asuu, *Ascaris suum*; Bmal, *Brugia malayi*; Cele, *Caenorhabditis elegans*; Crem, *Caenorhabditis remanei*; Hpol, *Heligmosomoides polygyrus*; Lloa, *Loa loa*; Mnig, *Mermis nigrescens*; Nbra, *Nippostrongylus brasiliensis*; Pdec, *Pseudoterranova decipiens*; Stra, *Syngamus trachea*; Tcan, *Toxocara canis*; Tcol, *Trichostrongylus colubriformis*; Tspi, *Trichinella spiralis*; Wban, *Wuchereria bancrofti*.
(TIF)

S2 Fig Examination of specific antisera against recombinant *Clonorchis sinensis* globins. The recombinant proteins (0.2 μg) resolved by 15% SDS-PAGE were transferred onto nitrocellulose membranes. After blocking with 5% nonfat dried milk diluted in TBST, the membranes were reacted with each of the specific antisera and then, horseradish peroxidase-conjugated rabbit anti-mouse IgG antibody. Positive reaction was visualized with an enhanced chemiluminescence detection system. For comparison, a gel image stained with Coomassie Brilliant Blue G-250 (CBB) is also presented.
(TIF)

S3 Fig Induction profiles of globin and hypoxia-inducible factor (*HIF*) genes in co-incubated *Clonorchis sinensis* adults and H69 cells. **A**. The live 56-day-old worms were co-incubated with the human cholangiocytes under the respective conditions. After 24 h incubation, the relative amounts of the *C*. *sinensis* gene transcripts were determined using qRT-PCR (n = 3, mean ± SD) and the fold increase in each of the experimental groups was calculated against that in the worm-only control group under 20% oxygen condition. **B**. Fold increase in the expression of *HIF-1α* and *2α* was similarly determined in the human cells. ^○^*P* < 0.05; ^☆^*P* < 0.01; ^★^*P* < 0.001; n.s., not significant.
(TIF)

S1 Data Numerical data for graphs in figures. Numerical data obtained from multiple technical replicates (Preps) in this study were contained in separate Excel spreadsheets. The mean and standard deviation (SD) of these measurements were calculated and used in the construction of graphs shown in Figs 3A, 4, 5A, 5B and 5C, 6A and 6B as well as S3A and S3B Fig.
(XLSX)

S1 Table Primers used in this study. (DOCX)
