# Supplementary figures and images for: Hypoxic and nitrosative stress conditions modulate expression of myoglobin genes in a carcinogenic hepatobiliary trematode, Clonorchis sinensis
Source: PLoS Negl Trop Dis. 2021 Sep 30;15(9):e0009811. doi: 10.1371/journal.pntd.0009811 (PMC8483323; doi:10.1371/journal.pntd.0009811)

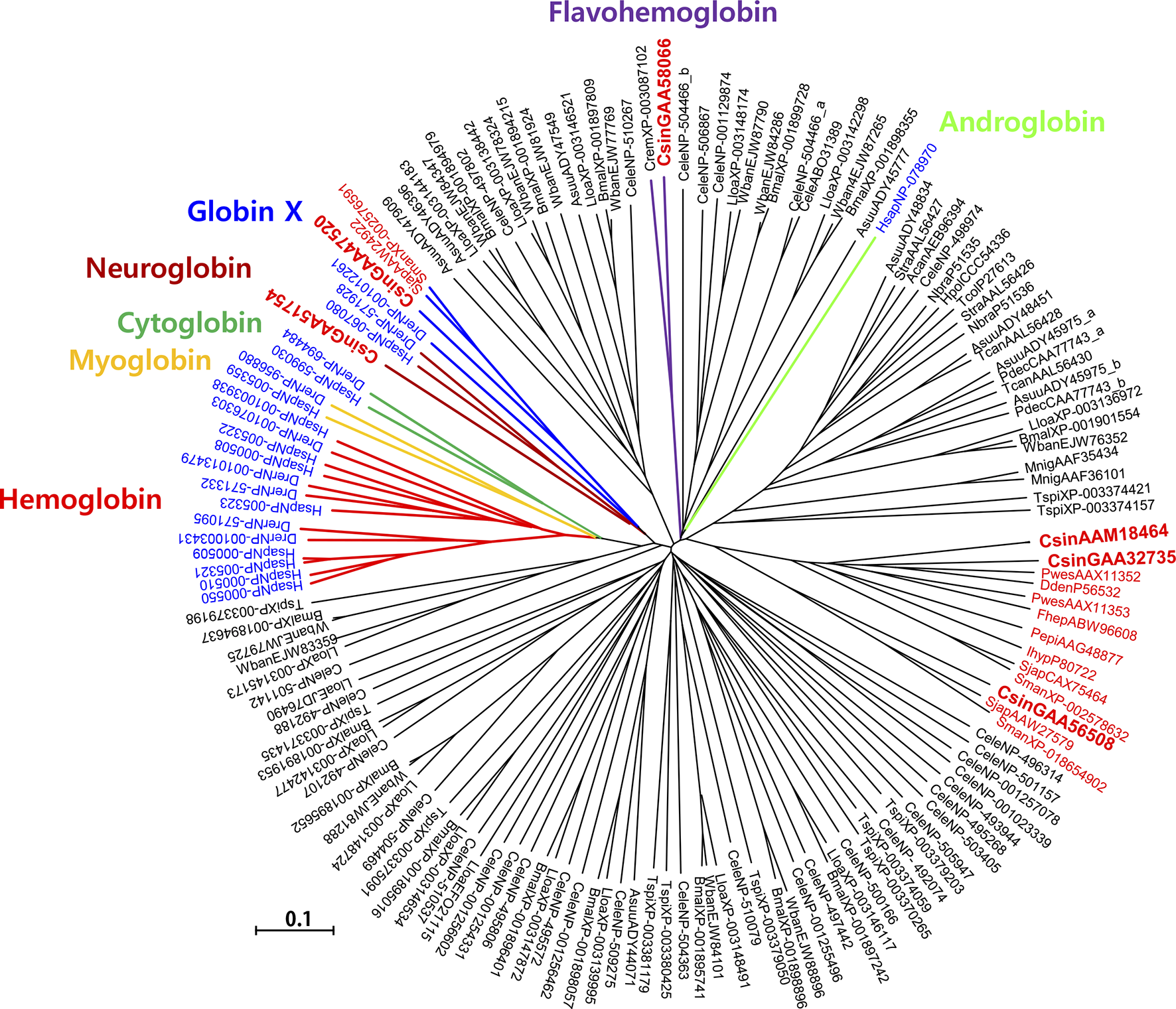

Supplement: S1 Fig — The trematode (red) and nematode (black) sequences were retrieved from the GenBank databases during similarity searches of Clonorchis sinensis globins using the BLAST programs. Sequences of human (Homo sapiens; Hsap) and zebrafish (Danio rerio; Drer) globins (blue) were also targeted in the analyses. The tree was constructed using Clustal X during the alignment of the retrieved sequences. The branches connecting members of each globin subfamily are differentiated from one another by colors identical to those of the corresponding subfamily names. The C. sinensis proteins are highlighted in boldface. Csin, C. sinensis; Dden, Dicrocoelium dendriticum; Fhep, Fasciola hepatica; Ihyp, Isoparorchis hypselobagri; Pepi, Paramphistomum epiclitum; Pwes, Paragonimus westermani; Sjap, Schistosoma japonicum; Sman, Schistosoma mansoni; Acan, Angiostrongylus cantonensis; Asuu, Ascaris suum; Bmal, Brugia malayi; Cele, Caenorhabditis elegans; Crem, Caenorhabditis remanei; Hpol, Heligmosomoides polygyrus; Lloa, Loa loa; Mnig, Mermis nigrescens; Nbra, Nippostrongylus brasiliensis; Pdec, Pseudoterranova decipiens; Stra, Syngamus trachea; Tcan, Toxocara canis; Tcol, Trichostrongylus colubriformis; Tspi, Trichinella spiralis; Wban, Wuchereria bancrofti. (TIF) [file pntd.0009811.s001.tif]

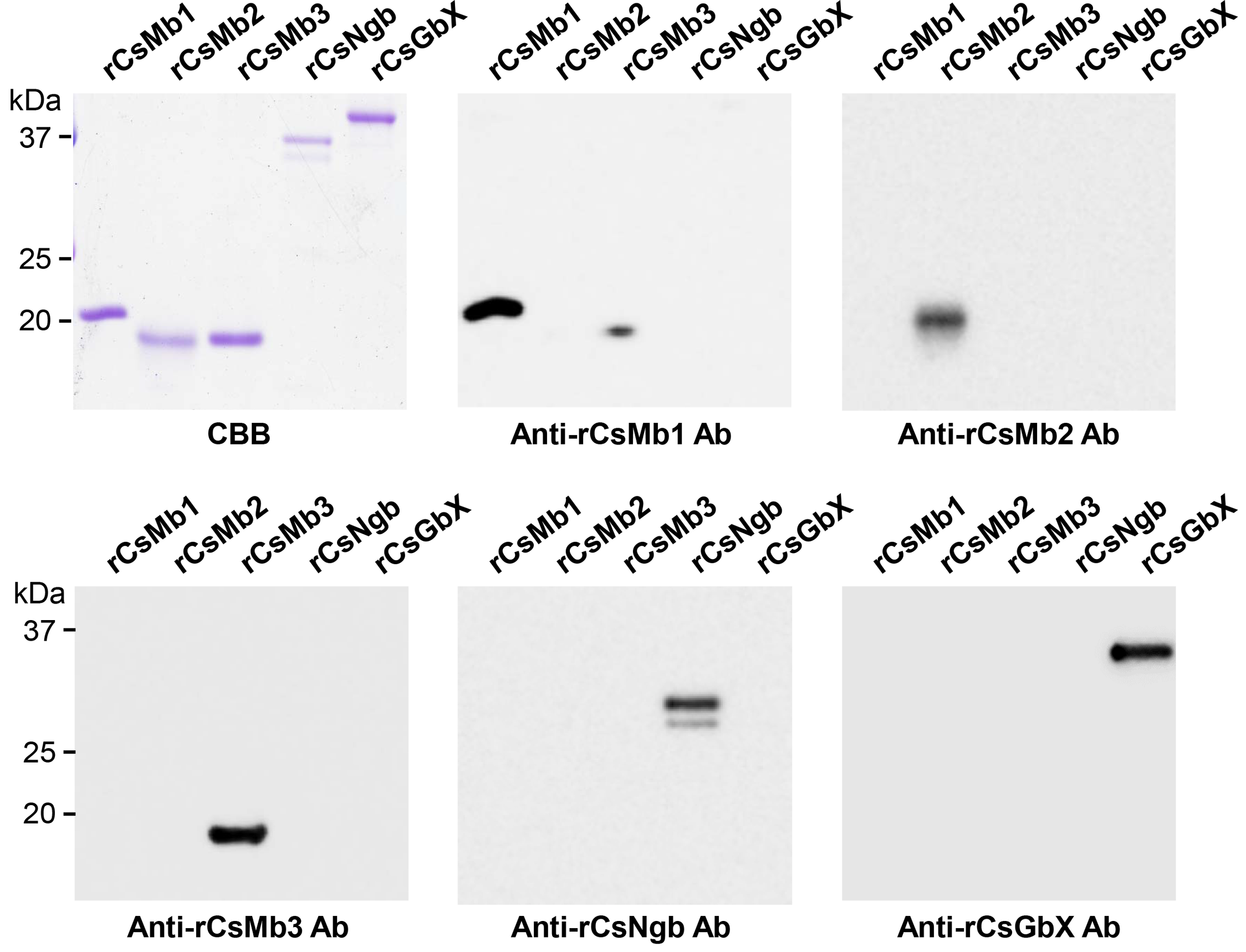

Supplement: S2 Fig — The recombinant proteins (0.2 μg) resolved by 15% SDS-PAGE were transferred onto nitrocellulose membranes. After blocking with 5% nonfat dried milk diluted in TBST, the membranes were reacted with each of the specific antisera and then, horseradish peroxidase-conjugated rabbit anti-mouse IgG antibody. Positive reaction was visualized with an enhanced chemiluminescence detection system. For comparison, a gel image stained with Coomassie Brilliant Blue G-250 (CBB) is also presented. (TIF) [file pntd.0009811.s002.tif]

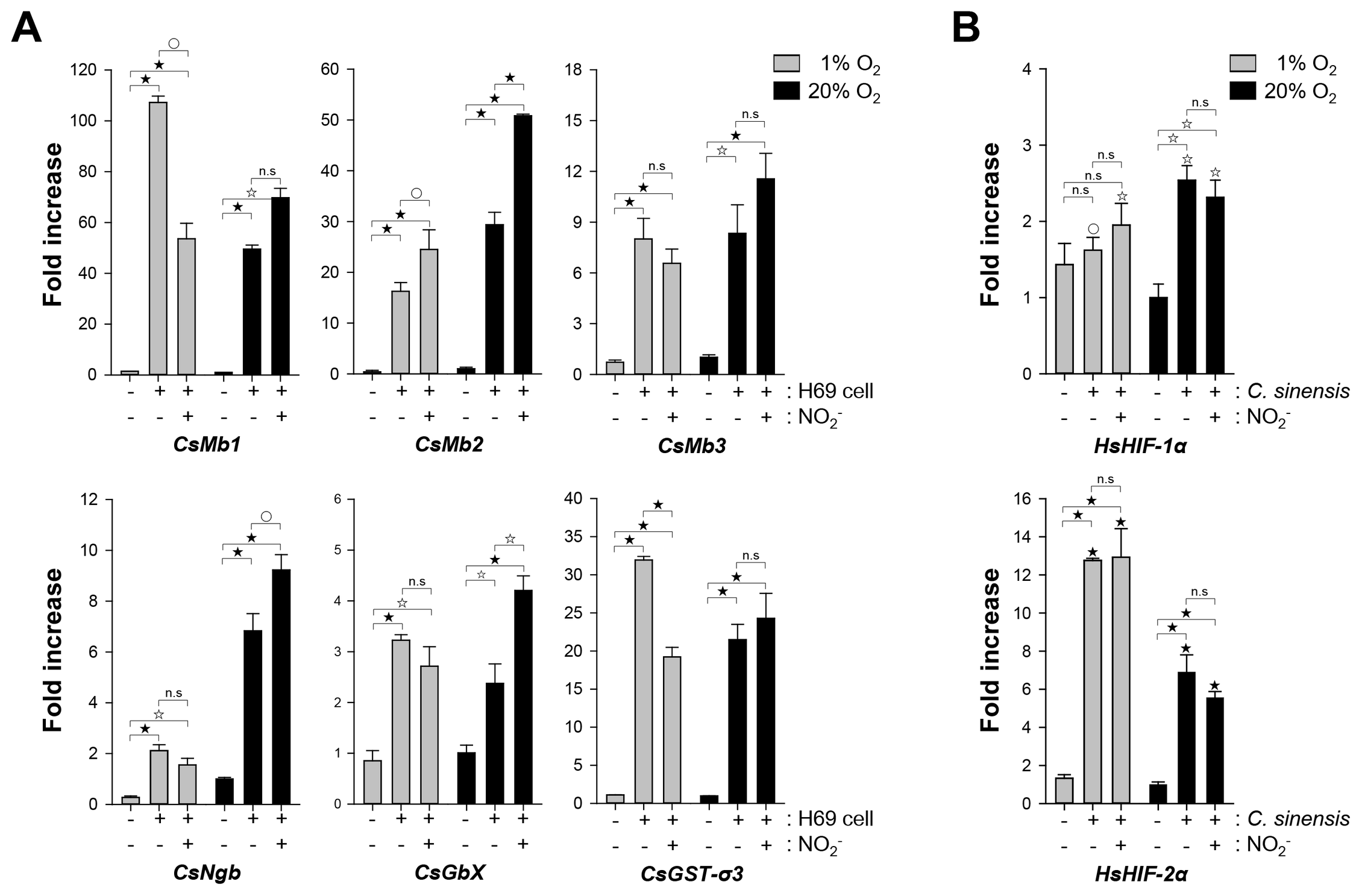

Supplement: S3 Fig — A. The live 56-day-old worms were co-incubated with the human cholangiocytes under the respective conditions. After 24 h incubation, the relative amounts of the C. sinensis gene transcripts were determined using qRT-PCR (n = 3, mean ± SD) and the fold increase in each of the experimental groups was calculated against that in the worm-only control group under 20% oxygen condition. B. Fold increase in the expression of HIF-1α and 2α was similarly determined in the human cells. ○P < 0.05; ☆P < 0.01; ★P < 0.001; n.s., not significant. (TIF) [file pntd.0009811.s003.tif]
