# Supplementary material for: Hypoxic and nitrosative stress conditions modulate expression of myoglobin genes in a carcinogenic hepatobiliary trematode, Clonorchis sinensis
Source: PLoS Negl Trop Dis. 2021 Sep 30;15(9):e0009811. doi: 10.1371/journal.pntd.0009811 (PMC8483323; doi:10.1371/journal.pntd.0009811)
Supplement: S1 Table — (DOCX) [file pntd.0009811.s005.docx]

**S1 Table.** Primers used in this study.

|  | **Gene name** | **Oligo name**^a^ | **Sequence (5′ → 3′)** |
| --- | --- | --- | --- |
| Amplification of full coding DNA sequences for gene cloning | | | |
|  | *CsMb1* | CsMb1-c-EF | GGGAATTCATGGCACCCCTATCAAAAGACG |
|  |  | CsMb1-c-XR | CCCTCGAG TTAGCCAAGAAAGCCGGCAAC |
|  | *CsMb2* | CsMb2-c-EF | GGGAATTCATGGCTCCGTTGACAGAGTCACAG |
|  |  | CsMb2-c-XR | CCCTCGAGCTAAAGGTCCTTTGAAATATTTGG |
|  | *CsMb3* | CsMb3-c-EF | GGGAATTCATGGATCCCAAACGACATAAGTC |
|  |  | CsMb3-c-XR | GGCTCGAGTCACAACCTCTTTCCAATCATTG |
|  | *CsNgb* | CsNgb-c-BF | AAGGATCCATGGGTGTGGAGCAAAGTCGAC |
|  |  | CsNgb-c-HR | GGAAGCTTTTATTTACACACAGAATAGAAGTGC |
|  | *CsGbX* | CsGbX-c-EF | GGGAATTCATGCAAACCAATGAACAATGTCC |
|  |  | CsGbX-c-XR | AACTCGAGCTACGACCTTCGTAGAGACCG |
|  | *CsFHb* | CsFHb-c-EF | AAGAATTCATGAGTAACCAGCGTAATGGCG |
|  |  | CsFHb-c-XR | AACTCGAGTTACAGCACCTTATGCGGGCC |
| Amplification of mRNA transcripts for quantitative RT-PCR analysis | | | |
|  | *CsMb1* | CsMb1-q-F | GGAGAAAAGTGGCAAAGAGC |
|  |  | CsMb1-q-R | CTTTTGTTAGCACTCCGCTG |
|  | *CsMb2* | CsMb2-q-F | GACAGAGTCACAGATTGCTG |
|  |  | CsMb2-q-R | CAGGATGTGCTCCAAGAAAG |
|  | *CsMb3* | CsMb3-q-F | TCACCACAACCGTCGAAGAA |
|  |  | CsMb3-q-R | GAGCCTGAGGAAGTGTCATTG |
|  | *CsNgb* | CsNgb-q-F | GAAAAGTTGATGGTGCTGGGAG |
|  |  | CsNgb-q-R | CGGCGAACATCCTCGGTAA |
|  | *CsGbX* | CsGbX-q-F | GAACAATGTCCTCGTTCCCT |
|  |  | CsGbX-q-R | TTATCTCCGCTTGGCTGAAG |
|  | *CsFHb* | CsFHb-q-F | GTGTTGGTCAAACGCCAATG |
|  |  | CsFHb-q-R | GACTGCCCCAGTTCCTTAAC |
|  | *β-actin* | CsbAct-q-F | CAAAGCAGGTTTTGCTGGTG |
|  |  | CsbAct-q-R | ATACCTCGTTTGGATTGGGC |
|  | *HsHIF-1α* | HsHIF1-q-F | TGAACATAAAGTCTGCAACATGGA |
|  |  | HsHIF1-q-R | TGAGGTTGGTTACTGTTGGTATCATATA |
|  | *HsHIF-2α* | HsHIF2-q-F | TGCTCCCACGGCCTGTAC |
|  |  | HsHIF2-q-R | TTGTCACACCTATGGCATATCACA |
|  | *HsRPL19*^b^ | HsRPL19-q-F | ATCATCCGCAAGCCTGTG |
|  |  | HsRPL19-q-R | TGACCTTCTCTGGCATTCG |

^a^E, *Eco*R I; X, *Xho* I; B, *Bam*H 1; H, *Hind* III.

^b^RPL19, Mitochondrial ribosomal protein L19 (house-keeping control gene for human cell).
